# The importance of academic teaching competence for the career development of university teachers: A comment from higher education pedagogy

**DOI:** 10.3205/zma001125

**Published:** 2017-10-16

**Authors:** Marianne Merkt

**Affiliations:** 1 Hochschule Magdeburg-Stendal, Zentrum für Hochschuldidaktik und angewandte Hochschulforschung, Magdeburg, Germany

**Keywords:** Academic teaching skills, continuing education in academic teaching, competency standards, assessing teaching skills

## Abstract

This contribution to the discussion focuses on which conditions at universities need to be established so that academic teaching skills become relevant to the career of university teachers. To find an answer, current findings on academic teaching are summarized from the literature.

## Zusammenfassung

Der hochschuldidaktisch fokussierte Diskussionsbeitrag beleuchtet die Frage, welche Bedingungen an Hochschulen und Universitäten entwickelt werden müssten, damit die akademische Lehrkompetenz für die Karriere von Hochschullehrenden relevant wird. Zur Beantwortung werden aktuelle Erkenntnisse aus dem hochschuldidaktischen Diskurs zusammengefasst.

## 1. Introduction

While continuing education aimed at developing academic teaching skills among university instructors is relatively systematic and widely established in Germany, the careers of university teachers are still almost exclusively defined by their research achievements. Several tentative approaches to also account for teaching competence – for instance in academic career negotiations – represent an exception, even though the relevance of teaching skills to the quality of a university degree program is no longer questioned. Medicine and the other health-related disciplines fit seamlessly into this context. The purpose of developing academic teaching skills is to enable teachers to design and shape high-quality learning experiences for future physicians and other professionals working in healthcare. This ability to engage in teaching refers not only to the individual level in specific class sessions and testing situations, but also to the organizational level of designing modules, study programs, creating assessments for study programs, and the related aspects such as study reform processes, quality management in teaching, e-learning, etc. Since a key feature of academic teaching is that it must be continually developed in alignment with research, and professional expertise must also be constantly honed, this makes the content and knowledge required in academic teaching dynamic. Teaching skills also encompass the design and development of competency-based curricula. The requirements to be met by academic teaching are just as demanding.

## 2. Development of academic teaching skills through continuing education and training

It is empirically proven that academic teaching skills require longer periods of time to develop. The reason for this lies in the action-guiding function of teaching/learning philosophies and teaching/learning concepts which are anchored emotionally since they affect perceived ideals and norms – the very role of the teacher, for example – as well as habitualized educational concepts [1]. This is why training sessions based on methodology alone are less sustainable because they are limited to the teacher’s performance. Another empirically confirmed factor in the development of pedagogical competency is the teaching/learning culture within a given circle of colleagues [2]. The application of newly acquired skills to daily teaching practices depends critically on the openness of the work environment and the support from colleagues for innovation.

## 3. Assessment of academic teaching skills

Also critical to a career is how academic teaching skills are assessed and what value and recognition they are given [3], [4].

Strategies have been developed and implemented in the neighboring European countries. In Great Britain a competency standard for teaching – the Professional Development Framework (PDF) [https://www.seda.ac.uk/pdf] – has been developed according to the basic career stages. This competency-based standard is the guideline for assessing teaching skills at different skills levels with differentiation in special areas such as e-learning, advising, quality management, etc. The defined competency levels in the PDF have been drawn upon as pre-requisites for hiring teachers in higher education. Teachers must demonstrate proper qualification in order to be hired. The lowest level is defined by the teaching skills of student tutors and mentors, ascending to those who are qualified to lecture at universities. The next level is for university teachers and instructors, doctoral candidates, and goes on to the highest level which applies to the appointment of professors. The PDF has been extensively adopted by Scandinavian and Dutch institutions of higher education.

A best-practice example taken from publications is the assessment procedure for hiring followed by the University of Lund’s School of Engineering Sciences. The assessment of a candidate’s teaching skills according to the PDF standard counts for 50% in the hiring process, meaning that it is equal to the assessment of the candidate’s research achievements. During the assessment procedure a candidate is observed while teaching two classes and is required to submit extensive documentation of his or her teaching qualifications. The commission exists of members of the school’s pedagogical academy. Belonging to this academy are teachers who not only can demonstrate teaching expertise, but who also engage in education research and have published in this area. Acceptance into the academy is by application and subject to acceptance. Academy members receive extra pay, but are also obligated to fulfill specific duties, such as participation on the assessment commissions. It is conceivable that the establishment of such a research-oriented academy substantially raises the reputation and recognition of the quality of the academic teaching and learning [5].

## 4. Conclusion

The development of pedagogical competency in the institutional context of higher education has multiple dimensions. These include individual, person-specific development along a career path. Another dimension is the institutional anchoring of pedagogical competence through continuing education and training programs, assessments, and the development of cultural contexts promoting quality. The third dimension, not discussed in depth here, involves the laying down of legal and procedural frameworks regarding teacher aptitude in the relevant statutes governing institutions of higher education and the regulations covering professorships, post-doctoral programs and doctoral programs. A fourth dimension is the development of competency-based national standards to which the universities are bound. Some strategies and approaches are visible among the German universities. However, in comparison to the developments seen in the neighboring European countries, primarily to the north and west, German universities have been unable to keep up.

## Competing interests

The author declares that she has no competing interests. 
